# Synthesis and characterization of phenylboronic acid-containing polymer for glucose-triggered drug delivery+

**DOI:** 10.1080/14686996.2019.1700394

**Published:** 2019-12-20

**Authors:** Guihua Cui, Kunming Zhao, Kewei You, Zhengguo Gao, Toyoji Kakuchi, Bo Feng, Qian Duan

**Affiliations:** a Center for Biomaterials, Jilin Medical University, Jilin, China; b Department of Materials Science and Engineering, Changchun University of Science and Technology, Changchun, Jilin, China; c Department of Research, Redpharm Biotechnology Co., Ltd, Beijing, China; d Chemical and Engineering College, Yantai University, Yantai, Shandong, China; e Division of Biotechnology and Macromolecular Chemistry, Graduate School of Engineering, Hokkaido University, Sapporo, Japan; f Department of Pharmacy, Jilin Medical University, Jilin, China

**Keywords:** Poly(*N*-isopropylacrylamide) (PNIPAM), D-glucosamine (GA), poly(3-﻿acrylamido- phenylboronic acid)(APBA), ﻿lower critical solution temperature(LCST), 103 Composites, 301 Chemical syntheses / processing, 501 Chemical analyses

## Abstract

Thermo-, pH- and glucose-responsive polymeric nanoparticles are of great interest in developing a self-regulated drug delivery system. The novel core-shell nanoparticles were synthesized by self-assembly of a phenylboronic acid-based block copolymer poly-(*N*-isopropylacrylamide)-block-poly(3-acrylamidophenylboronic acid) (PNIPAM_136_-b-PAPBA_16_) and a fluorescent complex glucosamine-poly(*N*-isopropylacrylamide)/Eu(III) (GA-PNIPAM)/Eu(III) based on the cross-linking between PBA- and GA-containing blocks in this work. The nanoparticles can be tuned via thermo-induced collapse or glucose-induced swelling at appropriate pH and temperatures; they had an average kinetic radius was about 80nm, and which showed excellent fluorescence. MTT assays revealed the nanocarriers had no signiﬁcant cytotoxic response of the micelle when it was observed in the cell line over the concentration range from 0.1 to 1000 μg/ml at any exposure times.

## GRAPHICAL ABSTRACT

**Figure:**
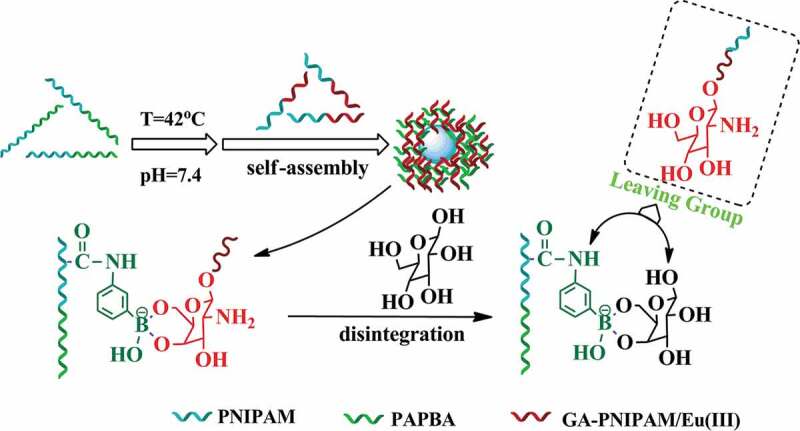

## 1. Introduction

Glucose-sensitive drug delivery system based on phenylboronic acid (PBA) has been a growing interest due to it can control the release of drugs automatically and continuously according to the change of glucose concentration [1,2]. PBA and its derivatives can be applied to glucose-sensitive drug delivery system for diabetes treatment in the future because of which can specific bind with 1.2-diols or polyols to formate reversible covalent PBA/diol complexes [3]. This specific binding property enables the PBA functionalized carrier to have good glucose sensitivity and makes PBA-based glucose-sensitive drug delivery from materials to medicine in the future [4,5]. The system may be a self-regulated drug control release system, it can reduce the number of insulin injections and improve the patient’s compliance. So PBA-based glucose-sensitive hydrogels, microgels and nano-carriers have made great progress [6–9]. However, there are many difficulties of PBA and its derivatives as the glucose-sensitive drug delivery system is used for diabetes treatment. For example, the pH of glucose response is higher than the physiological pH and glucose response concentration is much higher than the blood glucose level of diabetic patients [10]. Therefore, boronic acid-modified polymers can be coupled with other environment-sensitive monomers or polymers to synthesize multiple-responsive nano-carriers to be used in drug delivery, because these nano-carriers can undergo dissolution or self-assembly to achieve the drug release rate in response to the variational local environment through their functional groups. [11–13]. According to different response factors, PBA-based glucose-sensitive nano-carriers can be divided into pH response, temperature response, light response and other types.

Among these nano-carriers, the temperature response nano-carriers with special structure by using the temperature sensitivity of poly(*N*-isopropylacrylamide)(PNIPAM) can control the release of insulin by self-adjusting ‘on/off ’ mode and protect drugs from the degradation of enzymes, which have great potential application value in the treatment of diabetes [14–18]. Shi and co-workers prepared complex polymeric micelle (CPM) through the self-assembly of two types of diblock copolymers, poly(ethylene glycol)-*b*-poly(aspartic acid-co-aspartamidophenylboronic acid) (PEG-b-P(Asp-co-AspPBA)) and poly(*N*-isopropylacrylamide)-*b*-poly(aspartic acid-*co*- aspartamidophenylboronic acid) (PNIPAM-*b*-P(Asp-co-AspPBA)) [15]. The CPM enabled the repeated on–off release of insulin regulated by glucose level. To enhance the glucose sensitivity and self-regulated release of insulin, the group fabricated glucose-responsive polymer based on the complexation between a glucosamine (GA)-containing block copolymer PEG_45_-b-P(Asp-co-AspGA) and a phenylboronic acid (PBA)-containing block copolymer PEG_114_-b-P(Asp-co-AspPBA) with α-CD/PEG_45_ inclusion complex as the sacrificial template [15]. The cross-linking between PBA- and GA-containing blocks can enhance the stability of the shell. Therefore, the addition of a GA-containing block copolymer PEG-b-P (Asp-co-AspGA) resulted in the formation of core-shell-corona (CSC) complex micelles had a hydrophilic vesicular membrane which was favorable for the penetration of water-soluble substances and exhibited prominent glucose-responsiveness at physiological pH 7.4 [17,18]. With the development of nanotechnology, nanoscale drug carriers are attracting more and more attention [19,20].

**Scheme 1:**
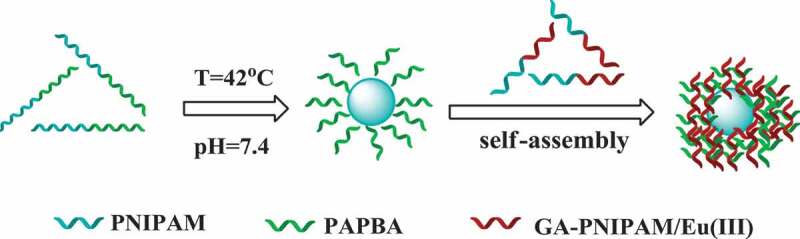
Schematic of the nanoparticles synthesis

In the previous research, most of the polymer preparation of GA used amino group for reaction, and most of the polymers were synthesized by free radical polymerization. However, in this study, we retained the amino of GA, which is better for retaining the activity of GA. We synthesized polymer by atom transfer radical polymerization (ATRP) and reversible addition-fragmentation chain transfer polymerization (RAFT), so the reaction is more controllable. Meanwhile, europium fluorescence system is adopted in this study, which can be directly used for determination in the future. In a previous work of our team, a series of thermo-sensitive GA terminated-PNIPAM polymers (GA-PNIPAM) had been developed in our team by ATRP [21,22], and which could coordinate with Eu(III) ions to form the (GA-PNIPAM)/Eu(III) complexes with amino. To explore the novel PBA-based glucose-sensitive drug delivery, in this work, the thermo-sensitive phenylboronic acid PBA-containing block copolymer poly(*N*-isopropylacrylamide)-block-poly(3-acrylamidophenylboronic acid) (PNIPAM_136_-b-PAPBA_16_) were fabricated by RAFT. Then, the addition of a GA-containing complexes (GA-PNIPAM)/Eu(III) resulted in the formation of core-shell complex micelles based on the cross-linking between PBA- and GA-containing blocks as shown in Scheme 1. The results of the transmission electron microscopy (TEM) and dynamic light scattering (DLS) revealed the complex micelles were excellent nanoparticles which may be a promising candidate for glucose-responsive drug delivery for diabetes treatment.

## 2. Materials and methods

### 2.1. Materials and instrumentation

*N*-Isopropylacrylamide (Aldrich, 98%) was recrystallized twice from a hexane/benzene mixture (3/2, v/v). Tris(2-(dimethylamino)ethyl)amine (Me_6_TREN) was synthesized from tris(2-amino-ethylamine (TREN, Aldrich, 99%) according to the literature [23]. CuBr (Aldrich, 99%) was washed successively with acetic acid and ether and then dried and stored under nitrogen. 3-Aminobenzeneboronic acid (Aladdin, 97%), acryloyl chloride (J&K Chemical, AR), 2,2ʹ-azobis(2-methylpropionitrile)(AIBN; J&K Chemical, 98%), 1,3,5-Trioxane (Aladdin, 99%), 1-dodecanethiol (Aladdin), tetrabutylammonium bromide (Aladdin), carbon disulfide (Aladdin), 2-bromo-2-methylpropionic acid (Acros, 98%), 2-bromopropionyl bromide(Aldrich, 97%), D(+)-glucosamine hydrochloride (Aladdin, 99%) and other chemical reagents were obtained commercially and were used as received unless otherwise stated. 3-[4,5-dimethylthiazol-2-yl]-2,5- diphenyltetrazolium (MTT) was purchased from Acros. Human hepatocellular carcinoma cells HepG2 in culture and NCTC clone 929 cells (L-929) were purchased from the Type Culture Collection of the Chinese Academy of Sciences, Shanghai.

The ^1^H nuclear magnetic resonance (NMR) spectra of monomers and polymers in CDCl_3_ were obtained on a Varian Unity 400 NMR spectrometer. Molecular weights (*M*_n_) and polydispersity (*M_w_*/*M*_n_) were measured using a gel permeation chromatograph (GPC), a Waters 510 pump and a Model 410 differential refractometer at 25°C. Tetrahydrofuran (THF) was used as the mobile phase at a ﬂow rate of 1.0 ml·min^−1^. The lower critical solution temperatures (LCSTs) of the polymer solutions were determined by absorbance at 500 nm, using a Shimadzu-2600 UV-Vis spectrophotometer with a heating rate of 0.1°C·min^−1^. The LCST was deﬁned as the temperatures corresponding to 10% decrease of transmittance. Polymer concentration was 1 mg/ml. The average particle size and size distribution of the nanoparticles were characterized by DLS with an Malvern470 instrument at a fixed scattering angle of 90°, after applying 0.45 *μ*m Millipore filters. The morphologies of the nanoparticles were studied by TEM using a JEOL JEM-2100 microscope. The cell viability was evaluated by MTT, the optical density (OD) was measured at 490 nm with a microplate reader (Bio-Rad, USA). Cell viability was determined as a percentage of the negative control (untreated cells).

### 2.2. General procedure for synthesis of nanoparticles

#### 2.2.1. Synthesis of PNIPAM-b-PAPBA

**Scheme 2:**
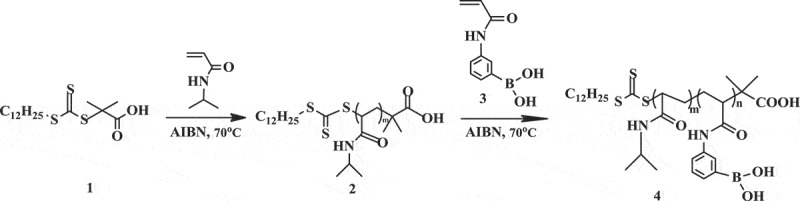
Synthesis of poly(3-acrylamidophenylboronic acid)-*b*-poly(*N*-isopropylacrylamide) (PNIPAM-b-PAPBA) via RAFT

Poly(*N*-isopropylacrylamide)-block-poly(3-acrylamidophenylboronic acid) was synthesized using NIPAM and 3-acrylamidophenylboronic acid by RAFT as shown in Scheme 2. Synthesis of block copolymers required four steps. Firstly, we synthesized *S*-1-dodecyl-*S*´-(*α,α*´-dimethyl-*α*´´-acetic acid) trithiocarbonate (DMP) (**1**) as a chain transfer agent (CTA). DMP was synthesized by a method derived from Lai et al. [24,25]. 1-Dodecanethiol (4.04 g, 20 mmol), tetrabutylammonium bromide (0.26 g,0.8 mmol), and acetone (10 ml) were mixed in a jacketed reactor with nitrogen gas for 30 min at 0°C. Sodium hydroxide solution (50%) (1.68 g, 21mmol) was added over 15 min. After stirred for an additional 15 min, carbon disulfide (1.525 g, 20mmol) in acetone (2.015 g, 10mmol) was added during which time the color turned red. chloroform (30 ml) was added in one portion after 15 min, followed by drop-wise addition of sodium hydroxide solution (50%)(8 g, 100mmol) over 30 min at 0°C. The reaction was stirred overnight at 25°C. Deionized water (30 ml) was added, followed by concentrated HCl (5 mL) to acidify the aqueous solution. The reactor was stirred intensely under a nitrogen atmosphere to help evaporate off acetone. The solid was collected with a Buchner funnel and then stirred in 2-propanol (100 ml). The undissolved solid was filtered to obtain **1** as a yellow solid (3.62 g, yield 49.7%, melting point 62.5°C) (the results are shown in Figures S1 and S2).

Secondly, we synthesized the macromolecular chain transfer agents **2**. A mixture of NIPAM (1.27 g, 11.2 mmol), DMP (40 mg, 0.112 mmol), 1,3,5-trioxane (50.44 mg, 0.56 mmol), AIBN (0.20 mg, 0.011 mmol) in anhydrous dimethylformamide (DMF, 5 ml) was sealed on middle side of an H-shaped ampoule glass and stirred, nitrogen was bubbled through both mixtures for 20 min to remove any oxygen. Three freeze-pump-thaw cycles were performed to degas the solutions. The ampoule was placed at 70°C. The samples were removed periodically by syringe to determine molecular weight. The polymerization was quenched by exposing the solution to air. The solution was concentrated under vacuum and the polymer was precipitated into cold ether three times and dried under a vacuum for 4 h to obtain **2** as a power (0.54 g, yield 42%, *M*_n_ = 7780g•mol^−1^) (the results are shown in Figures S3 and S4)﻿.

Thirdly, we synthesized 3-acrylamidophenylboronic acid(APBA) monomer **3**. The method was derived from Shinkai et al. [25,26]. 3-Aminophenylboronic acid (3.90 g, 21.9 mmol) was dissolved in a mixture of THF:H_2_O (66 ml, 1:1, v/v) in a 250-ml round-bottom flask. Sodium bicarbonate (4.05 g, 48.2 mmol) was added to the flask, and the mixture was cooled below 5°C. The mixture of acryloyl chloride (4.36 g, 48.3 mmol) in anhydrous THF (7 ml) was added drop slowly over 1 h. The reaction mixture was stirred overnight and allowed to reach room temperature. After THF was removed under vacuum, ethyl acetate (48 ml) was added into the solution then stirred for 2 h. The organic layers were washed with water twice and brine twice. Ethyl acetate was removed under vacuum, the resulting solid residue was added deionized water (42 ml), and heated at 80°C for 1h until a clear yellow solution was obtained. The solution was vacuum-filtered and cooled to below 10°C to obtain white power. The power was recrystallized by deionized water and dried in a vacuum oven at 25°C for 2 days to obtain **3** (1.53 g, yield 36.7%) (the results are shown in Figures S5 and S6).

Finally, we synthesized PNIPAM-b-PAPBA **4**. A mixture of APBA (0.383 g, 2.00 mmol), macro CTA (*M*_n_ = 7780 g•mol^−1^, 0.707 g, 0.091 mmol), 1,3,5-trioxane (50.44 mg, 0.56 mmol), AIBN (1.70 mg, 0.01 mmol) in anhydrous DMF:H_2_O (7.5 ml, 95:5, v/v) was sealed on middle side of an H-shaped ampoule glass and stirred, nitrogen was bubbled through both mixtures for 20 min to remove any oxygen. Three freeze-pump-thaw cycles were performed to degas the solutions. The ampoule was placed at 70°C for 1 h. The polymerization was quenched by exposing the solution to air. The solution was concentrated under vacuum and the polymer was precipitated into cold ether thrice. The product was dissolved in methyl alcohol and dialyzed for 48 h to purify it and was then dried under vacuum (0.76 g, yield 77%, *M*_n_ = 11100 g•mol^−1^)

#### 2.2.2. Synthesis of GA-PNIPAM/Eu(III) complexes

**Scheme 3:**
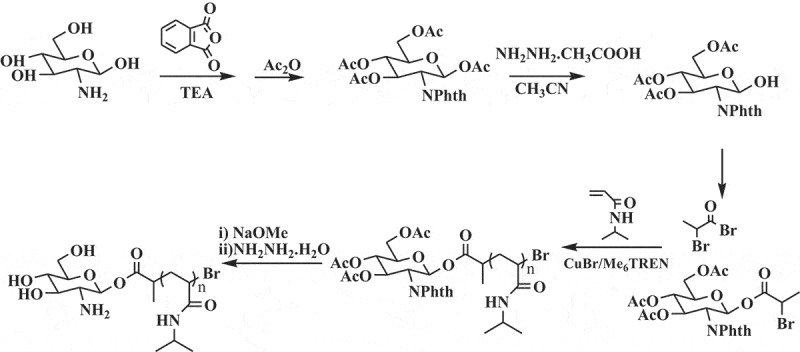
GA-PNIPAM synthesis by ATRP

The GA-PNIPAM/Eu(III) complexes had been synthesized previous work and the method derived from literature 19. The synthetic route of polymer GA-PNIPAM was shown in Scheme 3.

#### 2.2.3. Synthesis of [GA-PNIPAM/Eu(III)]–(PNIPAM-*b*-PAPBA) complex micelles

The block copolymers PNIPAM-b-PAPBA and the (GA-PNIPAM)/Eu(III) complexes were used for the preparation of the polymer nanoparticles in this study. PNIPAM_136_-b-PAPBA_16_(0.16g, *M*_n_ = 19200 g•mol^−1^, 0.8 mmol) was firstly dissolved in DMSO (l ml) for preparation of molecularly dispersed polymer solution. Then, it was diluted with PBS 7.4 solution with (GA-PNIPAM)/Eu(III) complexes (8 mmol) concentration of 0.8 mmol•ml for further studies. To prepare the nanoparticles, the solution was kept in a water bath at 42°C overnight to ensure the formation of the core–shell micelles owing to the complexation between PBA and GA, and the micelles with PNIPAM as shell and cross-linking PBA- and GA-containing blocks as core. Then, a given volume of (GA-PNIPAM)/Eu(III) complexes solution was added into the micellar solution under vigorous stirring according to the desired feed ratio. The solution was immersed in a water bath at 42°C for 24 h.

### 2.3. Biocompatibility study

Cell viability was investigated using NCTC clone 929 cells (L-929) and Human Hepatocellular Carcinoma Cells HepG2 in culture. After incubation for 24 h in 96-wells plates (8 × 10^4^ cells/ml per well) using Dulbecco’s modiﬁed Eagles medium (DMEM) in an incubator (37°C, 5% CO_2_), the culture medium was mixed with 200 μl of DMEM containing a sample of (GA-PNIPAM)/Eu(III) complexes, PNIPAM-b-PAPBA and [(GA-PNIPAM)/Eu(III)]–(PNIPAM-b-PAPBA) with a range of sample concentrations from 0.1 to 1000 μg/ml (according to the literature [21]): the mixture was further incubated for 48 h. Each sample was tested in six replicates per plate. Then, 20 µl of MTT solutions was added to the mixture in each well, which was incubated for additional 4 h. Next, 200 μl of DMSO was added, and the mixtures were shaken at room temperature. Six replicate wells were used for the control and test concentrations for each microplate. In addition, HepG2 cell suspension concentrations of 1 × 10^5^ cells/ml, 6 × 10^4^ cells/ml, and 4 × 10^4^ cells/ml were used the longer duration exposure experiments (24, 48, 72 and 96 h exposures, respectively) at each time-point of samples. The optical density was measured using a microplate reader at 570nm. The cell viability (%) was calculated according to the following Eq. (1):

(1) $$Cell\;viability\left(\%\right)=\left(A_{sample}/A_{control}\right)\times 100\%$$

where *A*_sample_ was the absorbance of the cells incubated in DMEM and mixture and *A*_control_ was the absorbance of the cells incubated in DMEM.

## 3. Results and discussion

### 3.1. Analysis of PNIPAM-b-PAPBA

**Figure 1.**
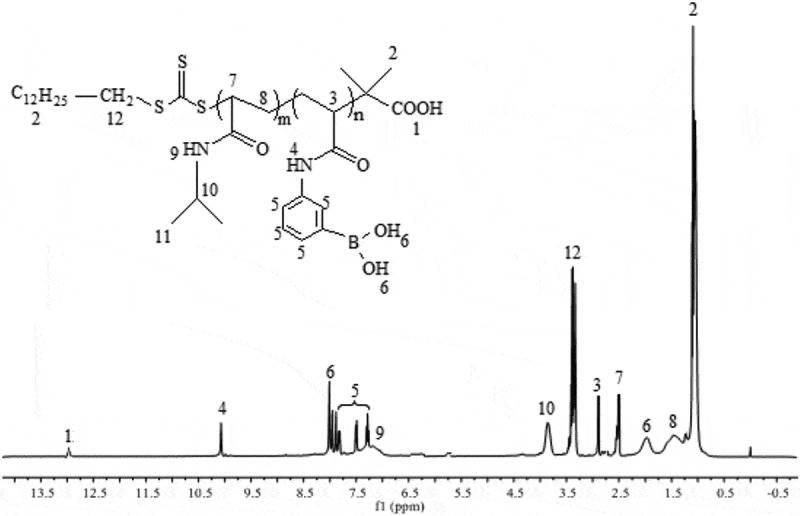
^1^H NMR spectra of PNIPAM-b-PAPBA

PNIPAM-b-PAPBA was synthesized by an APBA/macro-CTA/AIBN feed ratio of 20/1/0.1, which could obtain products having a narrow molecular weight distribution range of 1.22–1.39 as shown in Figure S7. The GPC traces of the block copolymer demonstrated a clean shift toward lower elution volume. The polymerization data of PNIPAM-b-PAPBA were shown in Table S1. The architecture of polymer was characterized by ^1^HNMR and IR spectroscopic analysis. Figure 1 shows the ^1^HNMR spectra of the PNIPAM-b-PAPBA, which showed all of the peaks expected for the PNIPAM and PAPBA blocks. Compared with macro-CTA (Figure S3), characteristic signals of PNIPAM representing methyl protons on isopropyl groups and protons adjacent to nitrogen atoms were shifted to 3.75 and 6.73 ppm, respectively. On the spectrum, the peaks at 7.93, 7.83, 7.56, 7.34, 8.00, 10.10 ppm confirmed the characteristic group of APBA.

**Figure 2.**
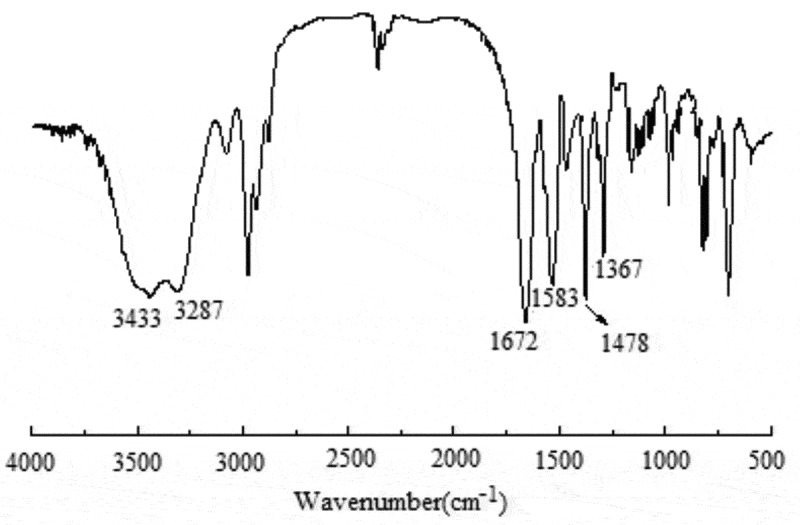
Fourier infrared transmittance spectrum (FT-IR) of PNIPAM-b-PAPBA

Figure 2 shows an Fourier-infrared transmittance spectrum (FT-IR) of PNIPAM-b-PAPBA. Compared with macro-CTA (Figure S4), the peaks at 1367 cm^−1^ were ascribed to stretching vibration of PBA group, and 1478 cm^−1^ were due to stretching vibration of benzene in APBA.

**Figure 3.**
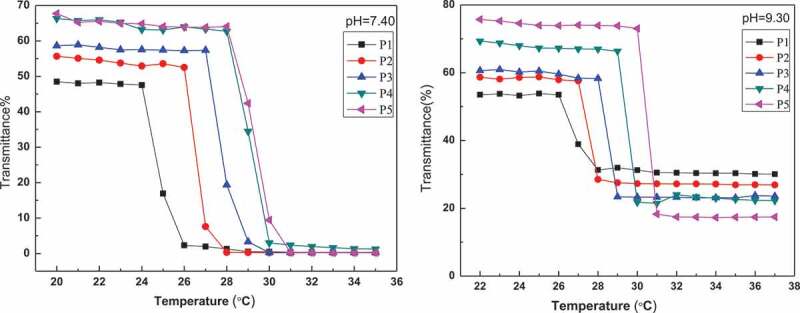
Transmittance of PNIPAM-b-PAPBA with different molecular weights at λ = 500 nm and for pH = 7.4 and 9.3

PBA-containing polymers have uniquely stimuli-responsive because their water solubility is determined by pH and solution diol concentration [27]. The p*K*_a_ value of PAPBA is about 9.3, and the value can be reduced by linking with diol [28]. To investigate the micelles based on the cross-linking between PBA- and GA-containing blocks, the thermo-responsive property of PNIPAM-b-PAPBA was studied by turbidimetry in two conditions (pH value of 7.4 and 9.3) respectively. Figure 3 shows the LCSTs of the PNIPAM-b-PAPBA at pH value of 7.4 and 9.3. The LCSTs are shown in Table 1. All LCSTs of PNIPAM-b-PAPBA were below 32°C (PNIPAM’s LCST); this was attribute to that hydrophobic PBA group was introduced into blocks polymers. It is expected that the pH value of the media has a great effect on the volume phase transitions of both micelles. From Table 1, the LCST of the same polymer in solution (pH value of 9.3) was higher than in solution (pH value of 7.4). The reason is that the pH value of 9.3 is exactly equal to p*K*a value of APBA; therefore, the APBA groups in the polymer is formated borate anion, which can enhance the hydrophilicity of polymer. The reason can be explained that the LCST increased with the increase of phenylboric acid content of the polymer. The LCST of P5 was closest to body temperature of above polymer, so it was used to prepare complex micelles in this article.

**Table 1.** LCST behavior of PNIPAM-b-PAPBA with different pH medium

	LCST(°C)	
Polymer	pH = 7.4	pH = 9.3
P1 (PNIPAM_25_-b-PAPBA_27_)	24.1	25.9
P2 (PNIPAM_66_-b-PAPBA_20_)	26.5	27.1
P3 (PNIPAM_94_-b-PAPBA_21_)	27.2	28.1
P4 (PNIPAM_128_-b-PAPBA_19_)	28.3	29.0
P5 (PNIPAM_136_-b-PAPBA_16_)	28.4	30.3

### 3.2. Analysis of [GA-PNIPAM/Eu(III)]–(PNIPAM-b-PAPBA) complex micelles

**Figure 4.**
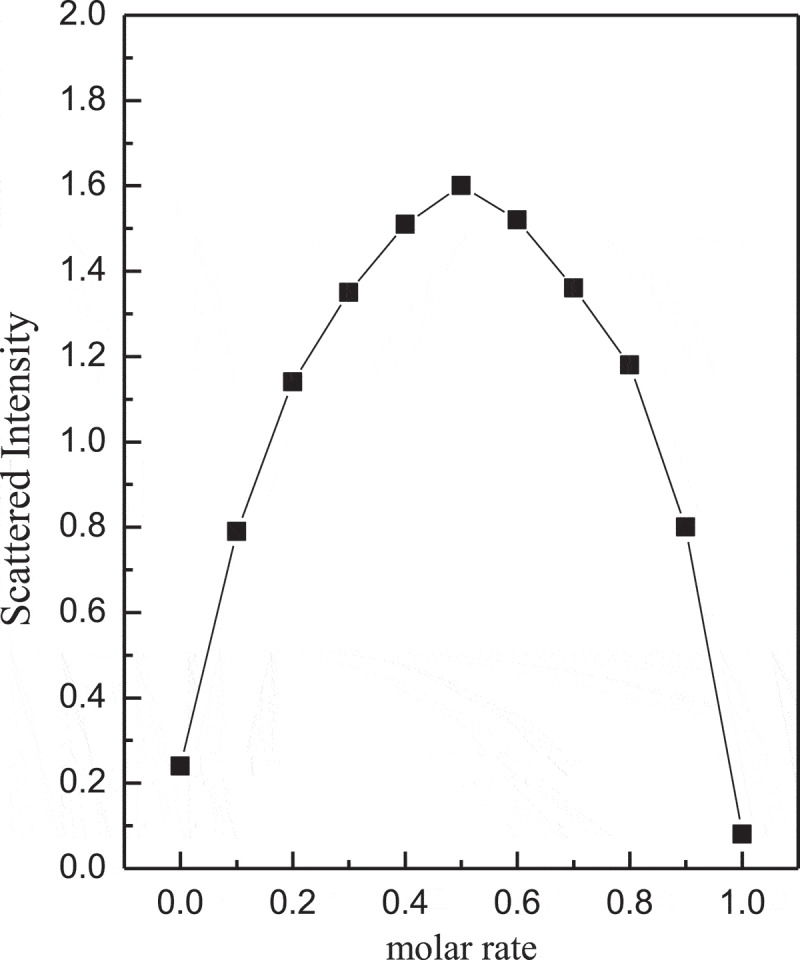
Scattered light intensity recorded for the aqueous mixture (pH = 7.4, T = 42°C)

The scattered light intensity of the complex micelles at different rates of (GA-PNIPAM)/Eu(III) and (PNIPAM-b-PAPBA) was used to study the dependence of complex micelles on substance concentration and the results are shown in Figure 4. The complex micelles had the maximum scattering intensity while the molar ratio of the PBA in PNIPAM-b-PAPBA and the GA in (GA-PNIPAM)/Eu(III) was 1:1. This result indicated that cross-linking occurred between the two mixtures and the larger aggregation was formed.

**Figure 5.**
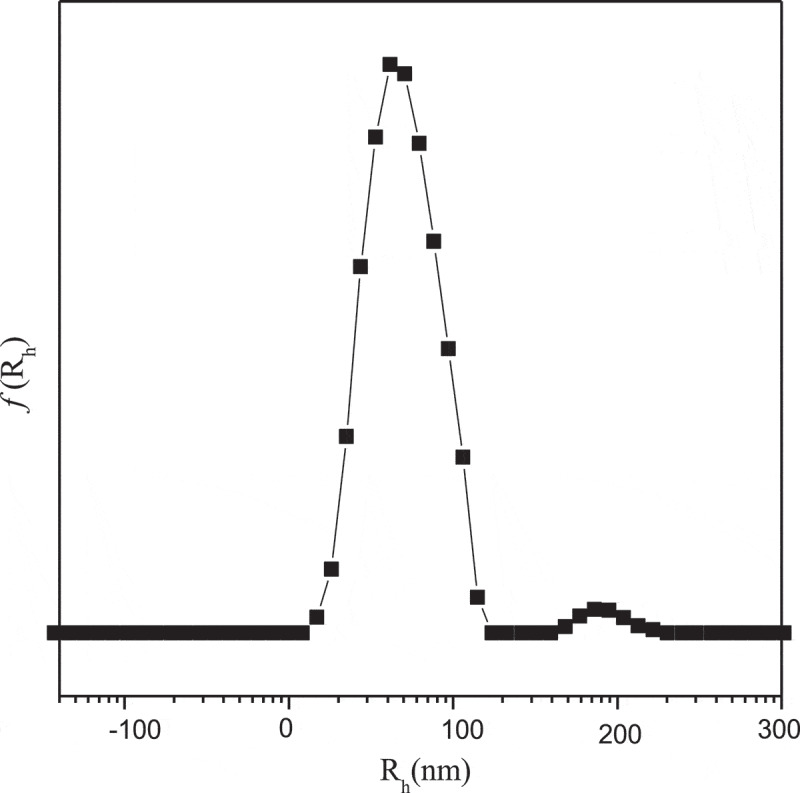
Intensity-average hydrodynamic radius distributions *f*(*R*_h_) of complex micelles

Figure 5 presents the intensity-average hydrodynamic radius distributions f(*Rh*) of complex micelles. The *Rh* was about 80nm, and which was calculated by Stokes–Einstein relation. There are few larger complex micelles near the average radius of 180 nm, which may be caused by the formation of GA-bis(boronate) complex which is more favorable in the ‘core’ area [29].

**Figure 6.**
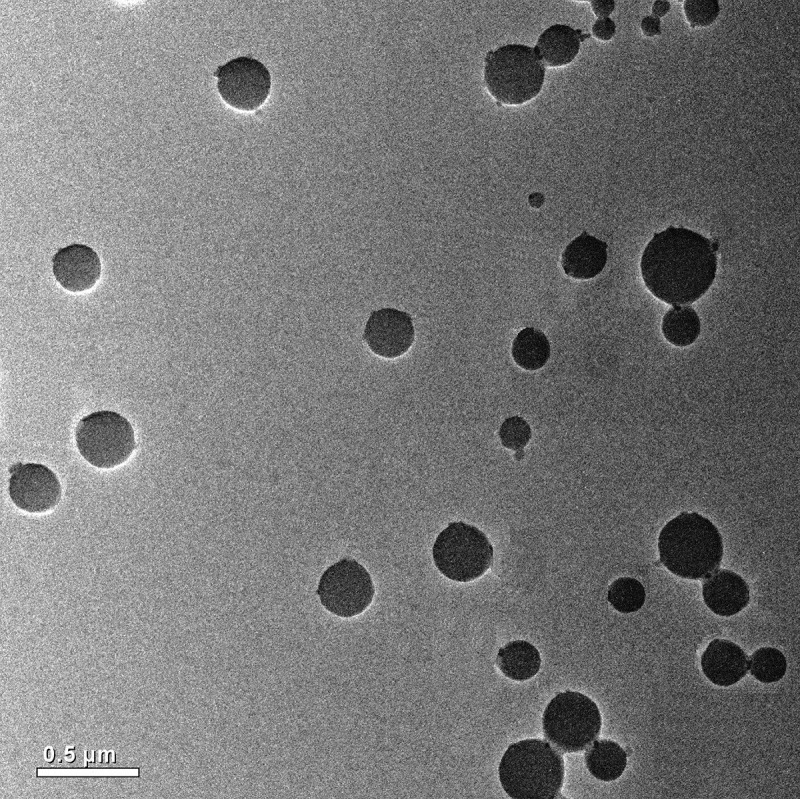
TEM images of self-assembly micelles (Scale bar 0.5 μm)

TEM images of the complex micelles provided information on size, shape, and distribution (Figure 6). Since no macroscopic phase separation occurred in the mixed system, we thought that the composite micelle was formed. Its core was composed of PAPBA/PAGA complex, and the soluble PNIPAM block was a shell. It can be seen from Figure 6 that the diameter of the complex micelles was about 130 ~ 380nm, which was larger than that of DLS test. It may be caused by the extrusion of the sample.

In order to confirm the presence of complex micelles as a result of cross-linking between PBA- and GA-containing blocks, this study adopts a ternary system. The glucose was added to the complex micelles. Glucose will connect with PAPBA to form a new covalent bond because of competitive complexation as shown in Scheme S1. The cross-linking between PBA- and GA of (GA-PNIPAM)/Eu(III) will be broken when the external glucose concentration increases, and a new covalent bond will be formed between PAPBA and glucose in system. After the newly formed micelle was dialyzed, no fluorescence of the new substance was found under the ultraviolet light. However, red fluorescence is observed from the dialysate (freeze-dried) in Figure S7. This phenomenon reveals that the GA of (GA-PNIPAM)/Eu(III) was separated from the cross-linked complex micelles. Uv-vis absorption spectra were used to further confirm that micelles were formed by the reversible cross-linked between PBA- and GA-containing blocks (Figure S8). The characteristic absorption peak of Eu(III) at 355nm is clearly seen of complex micelles in Figure S8. The characteristic absorption at 267nm is the peak of PBA of micelles, which is red shifted to 262 nm in the PNIPAM-b-PAPBA and glucose.

**Figure 7.**
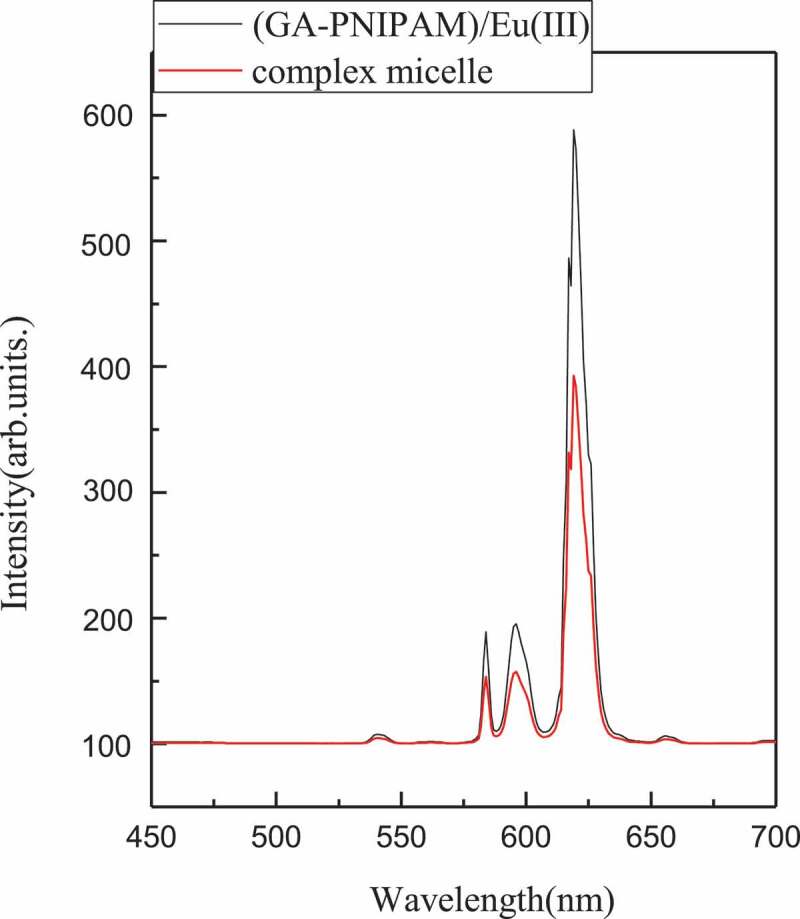
Fluorescence spectra recorded for (GA-PNIPAM)/Eu(III) and complex micelles (excitation wavelength 355 nm)

The fluorescence spectra of (GA-PNIPAM)/Eu(III) and complex micelle are shown in Figure 7. The complex micelle displayed four strong, narrow emission peaks at 579, 591, 613 and 658 nm, corresponding to the ^5^*D*_0_→^7^*F_J_*, (*J* = 0, 1, 2, 3) electronic transitions, respectively, which occurred from the excited *D* state to the multiplet *F* state. The most pronounced peak was at 613 nm (^5^*D*_0_→^7^*F*_2_). These are strong evidence for the formation of complex micelles which was the cross-linking between PBA- of PNIPAM-b-PAPBA and GA- of (GA-PNIPAM)/Eu(III). The fluorescence intensity of the composite micelle was lower than that of the (GA-PNIPAM)/Eu(III) with the same concentration, because of the lower content of europium.

**Figure 8.**
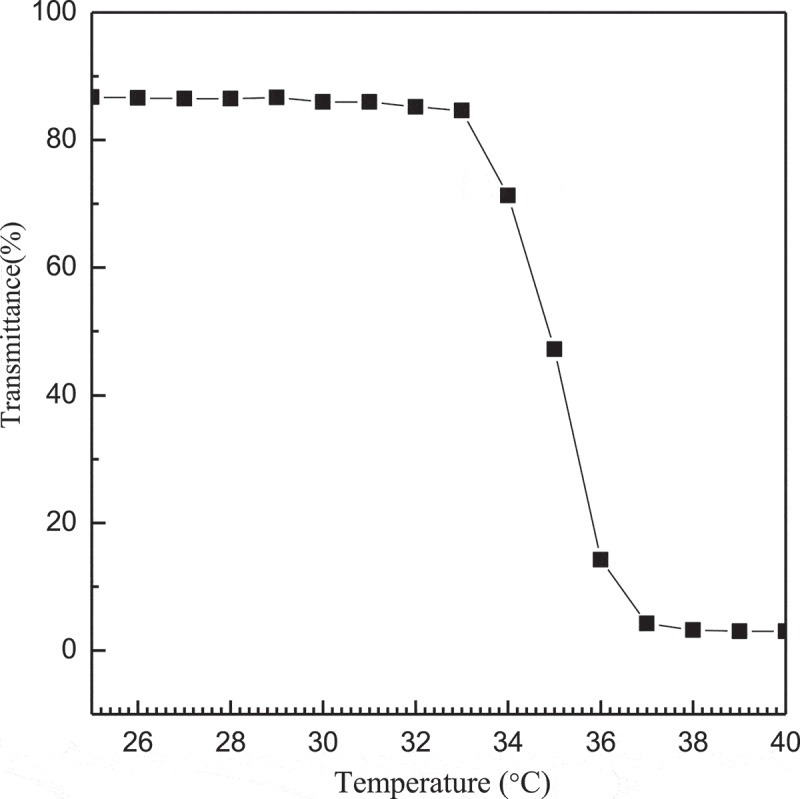
Transmittance for pH = 7.4 solutions of complex micelles

Thermo-responsive properties of the complex micelles will assist the carrier in drug release, so we studied the transmissivity of complex micelles with the change of temperature and the results are shown in Figure 8. It can be seen that the transmittance depends on temperature, and there is basically no change of the transmittance in the range of 20 ~ 33°C. When the temperature above 33°C, the transmittance of the complex micelles rapidly drops from 86.72% to 77.65%, and continues to rise until the transmittance of the complex micelles at 33.6°C drops to 44.2% and finally reaches a plateau. Therefore, it can be deduced that the LCST of the complex micelles is 33.6°C.

**Figure 9.**
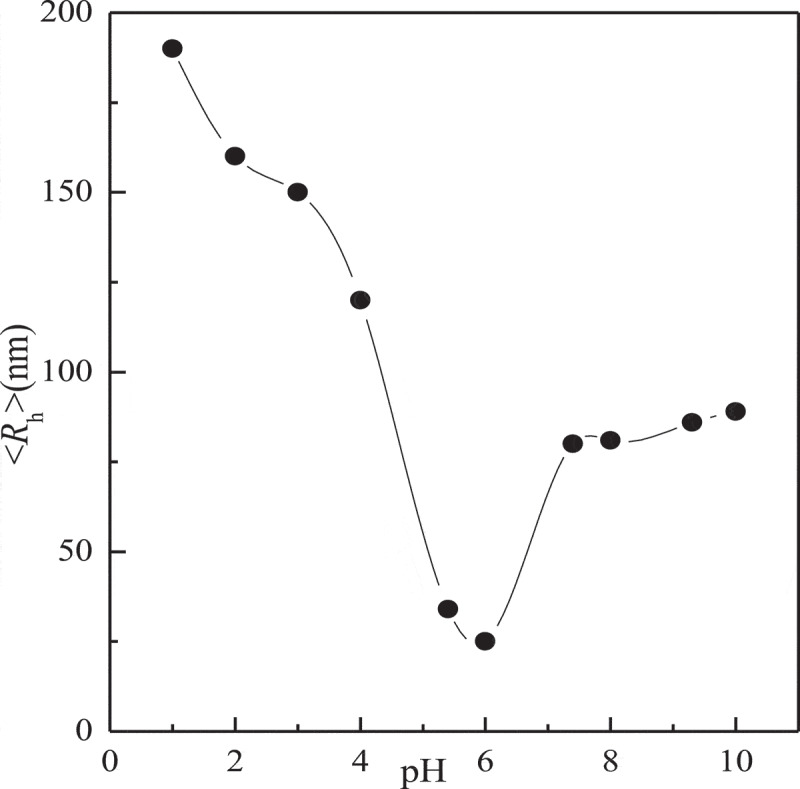
Ph dependence of scattered intensity-average hydrodynamic radius <*R*_h_> recorded for the aqueous mixture of complex micelles

**Figure 10.**
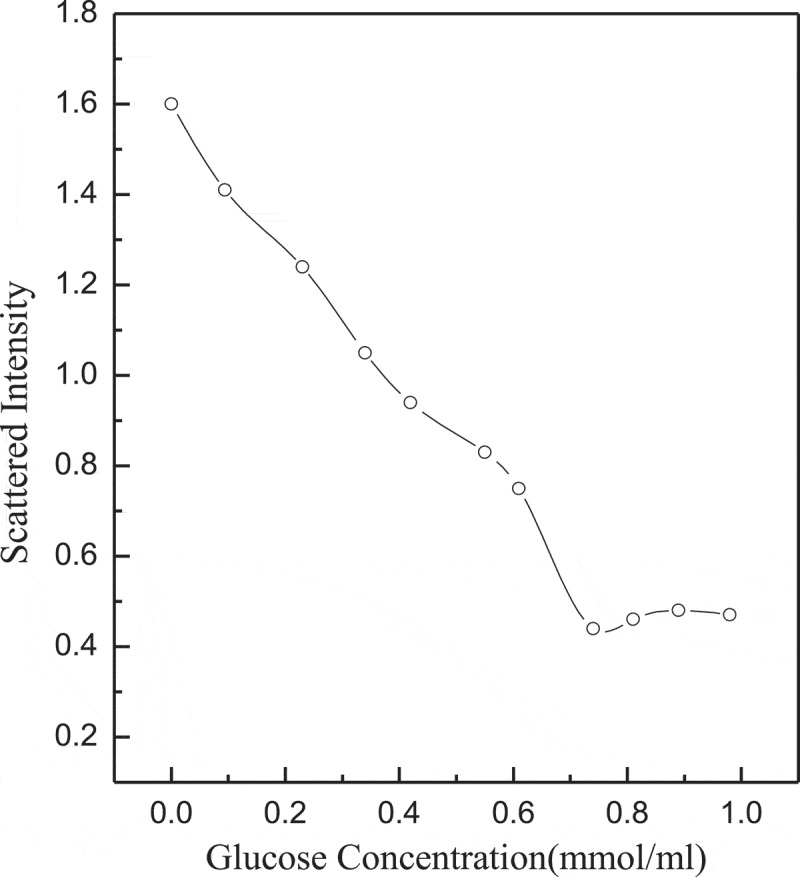
Glucose concentration dependence of scattered light intensity recorded for the aqueous mixture of complex micelles at pH = 7.4

The complex micelles are responsive not only to temperature but also to the pH and glucose concentration (Figures 9 and 10). From Figure 9, the fluid dynamics diameter of complex micelles is about 80nm at pH = 7.4. When the pH value is between 6 and 7.4, the hydrodynamic diameter of the complex micelles with the decrease of pH. This is due to the cross-linking between PBA- and GA-containing blocks towards the direction of dissociation when the pH value is less than p*K*_a_. PNIPAM-b-PAPBA is hydrophobic and will form a micelle with PAPBA as the core and PNIPAM as the shell, the diameter rather than cross-linked between PBA- and GA-containing blocks.

Figure 10 shows the scattering light intensity of complex micelles to glucose concentration. The *pK*_d_ (dissociation constant) of boronic acid is about 9.3, so PNIPAM-b-PAPBA and (GA-PNIPAM)/Eu(III) exhibit the strongest complexation at pH value of 9.3. In order to study its biological properties, this study takes pH value of 7.4 as an example. The scattering light intensity was decreased with the increase of glucose concentration. The reason is the competition between GA-containing blocks and glucose, it can cause the complex micelles dissociation [25]. There is no aggregation of large particles in solution, which may result in a stronger generation of phenylborates.

### 3.3. Assessment of cell viability

**Figure 11.**
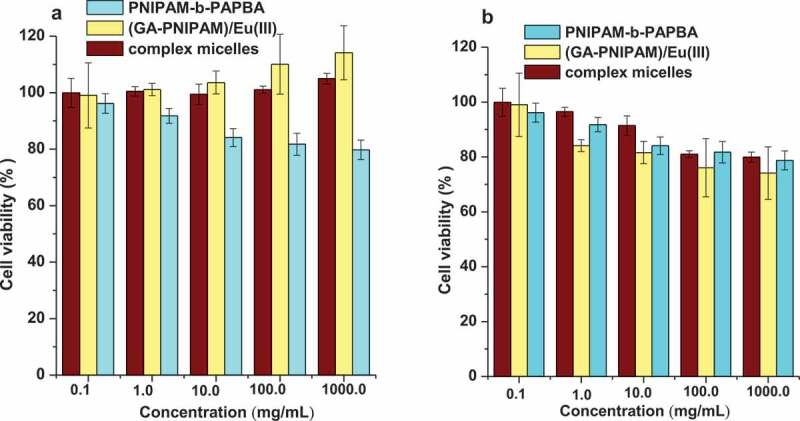
Viability of (a) the L-929 cells and (b) the MCF-7 cells incubated with the samples ((GA-PNIPAM)/Eu(III), (PNIPAM-b-PAPBA) and complex micelles), over a range of sample concentrations from 0.1 to 1000 μg/ml by MTT assay for 48 h

The (GA-PNIPAM)/Eu(III), (PNIPAM-b-PAPBA) and complex micelles were used to evaluate the cytotoxicity on L-929 and MCF-7(Figure 11). The percent of cell viability was determined by comparison with cells that were not exposed to samples, which were used as the control group. All three compounds were toxic in either cell line over a broad concentration range from 0.10 to 1000.00μg/ml. By introducing the hydrophilic monomer GA into the PNIPAM chain, the (GA-PNIPAM)/Eu(III) and complex micelles made the L-929 cells remained at a high level increasing over 100%, and the viability of the MCF-7 cells decreasing to approximately 80%.

## 4. Conclusions

The novel thermo-, pH- and glucose-responsive polymeric nanoparticles were fabricated by the cross-linking between PBA of PNIPAM-b-PAPBA and GA of GA-PNIPAM/Eu(III) to form complex micelles. The nanoparticles exhibited strong fluorescence and a fluid dynamics diameter approximately 80 nm at pH = 7.4. MTT assays revealed the nanocarriers had no signiﬁcant cytotoxic response. The nanoparticles will have potential applications for glucose-responsive drug delivery for diabetes treatment.

## Supplementary Material

Supplemental Material
