# Supplementary material for: Synthesis and characterization of phenylboronic acid-containing polymer for glucose-triggered drug delivery+
Source: Sci Technol Adv Mater. 2019 Dec 20;21(1):1–10. doi: 10.1080/14686996.2019.1700394 (PMC6968588; doi:10.1080/14686996.2019.1700394)
Supplement: Supplemental Material [file TSTA_A_1700394_SM1937.pdf]

Supplementary Information for

Synthesis and characterization of multiple-responsive phenylboronic acid-containing polymer

Guihua Cui[a,b], Kunming Zhao[a†], Kewei You[c], Zhengguo Gao[d], Toyoji Kakuchi[e], Bo Feng[f*], Qian Duan[*b]

[a] Center for Biomaterials, Jilin Medical University, Jilin,132013, China.

[b]Department of Materials Science and Engineering, Changchun University of Science and Technology, Changchun, Jilin, 130022, China.

[c] Redpharm (Changchun) Biotechnology Co., Ltd, Changchun, 130051, China.

[d]Chemical and Engineering College, Yantai University, Yantai, Shandong, 264005, China.

[e]Division of Biotechnology and Macromolecular Chemistry, Graduate School of Engineering, Hokkaido University, Sapporo, 060-8628, Japan.

[e]Department of Pharmacy, Jilin Medical University, Jilin 132013, China

*Correspondence: Bo Feng, cuiyuhan1981_0@sohu.com. Qian Duan, duanqian88@hotmail.com;

[†]These authors have contributed equally to this work.

Author Kewei You was employed by company Redpharm (Changchun) Biotechnology Co., Ltd. All other authors declare no competing interests.

Analysis of DMP(1).

Fig. S1 showed the ^{1}HNMR spectra of DMP. The peaks 3.30 ppm(-CH_2, 2H, between -$C_{12}H_{25}$ and -S-), 1.72ppm(-CH_3, 6H, near -COOH), 12.95ppm(-COOH, 1H).

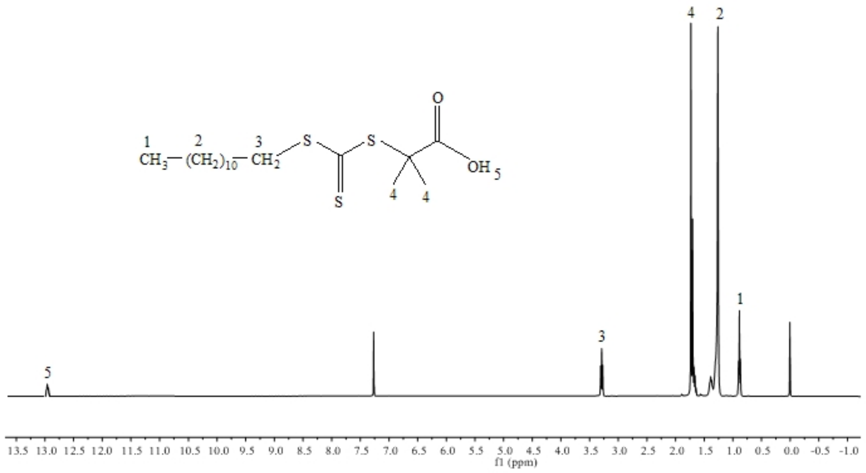

Fig. S1 ^{1}H NMR spectra of the DMP in $CDCl_3$.

Fig.S2 showed the FT-IR spectra of the DMP. We can observe the stretching vibrations -C-H(2920 cm^{-1}) , -CH_2 (2851cm^{-1}) -C=O (1719cm^{-1}), -COOH (1281cm^{-1}) , -C=S(1171cm^{-1}, 1070cm^{-1}), -C-S (812cm^{-1}), -CH_2-S(720cm^{-1}), respectively.

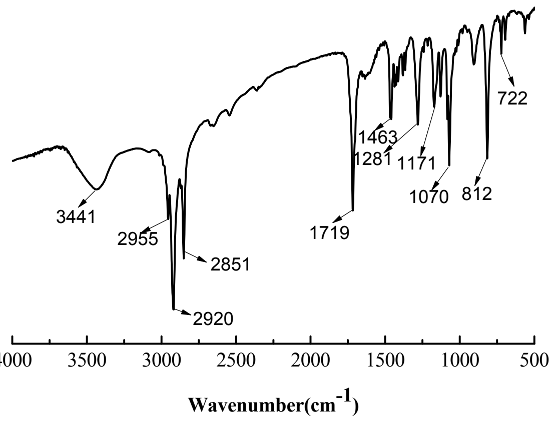

Fig. S2 FT-IR spectra of DMP.

Analysis of macromolecular chain transfer agents (2).

Fig. S3 showed the ^{1}HNMR spectra of macromolecular chain transfer agents. The characteristic signals of PNIPAM at 3.87 ppm and 7.29 ppm representing methyl protons on isopropylgroups and the characteristic signal of protons adjacent to nitrogen atoms, respectively.

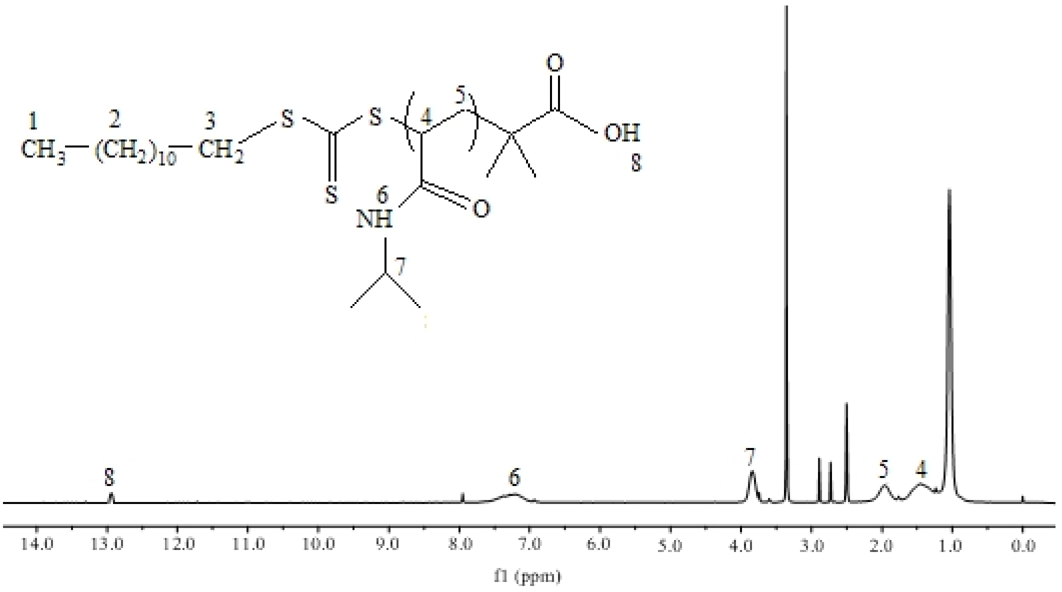

Fig.S3 ^{1}H NMR spectra of the trithiocarbonate-PNIPAM

Fig.S4 showed the FT-IR spectra of the macromolecular chain transfer agents. The band at 1643 cm^{-1} was ascribed to amide I [mainly the carbonyl stretching vibration ($\nu_{C=O}$)] and the band at 1544 cm^{-1} was ascribed to amide II [mainly the N–H bending vibration (δ_{N-H})].

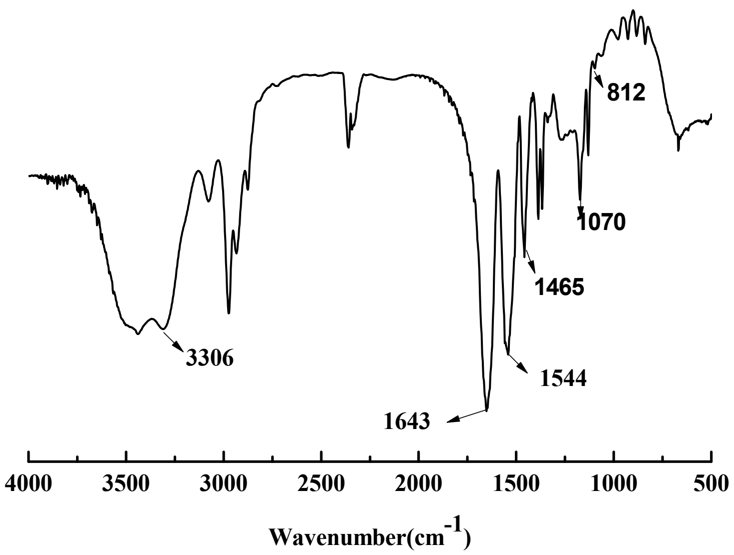

Fig. S4 FT-IR spectra of trithiocarbonate-PNIPAM

Analysis of APBA(3)

Fig. S5 showed the ^{1}HNMR spectra of the APBA. The peaks δ=10.07ppm (s, 1H,NH), 7.95ppm (s, 2H, $B(OH)_2$), 7.85, 7.80-7.78, 7.47-7.46, 7.27-7.24ppm (s, d, d, t, 1H each, ArH), 6.44-6.39, 6.23-6.19ppm (2d, dd, 1H each, vinyl CH 2), 5.71-5.69ppm (dd, 1H, vinyl CH).

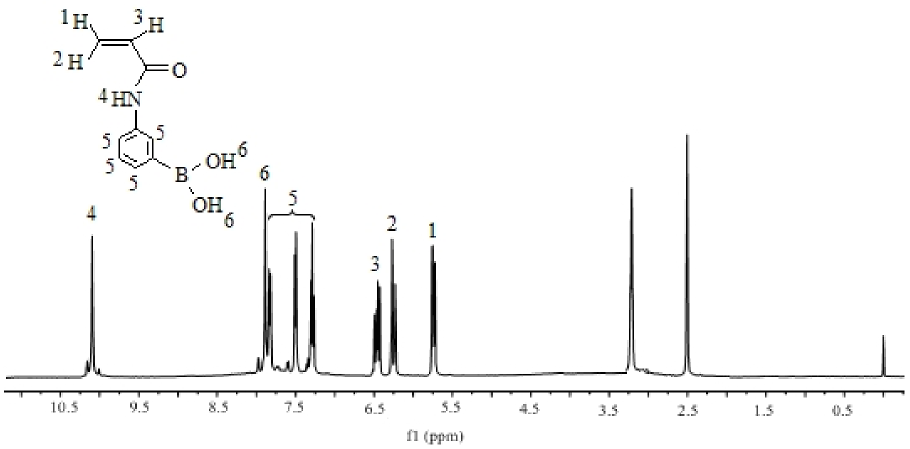

Figure S5 ^{1}H NMR spectra of APBA.

Fig.S6 showed the FT-IR spectra of the APBA. The band at 1663 cm^{-1} was ascribed to amide I [mainly the carbonyl stretching vibration ($\nu_{C=O}$)] and the band at 1558 cm^{-1} was ascribed to amide II [mainly the N–H bending vibration (δ_{N-H})]. The peak at 1351 cm^{-1} was stretching vibration of Boric acid group.

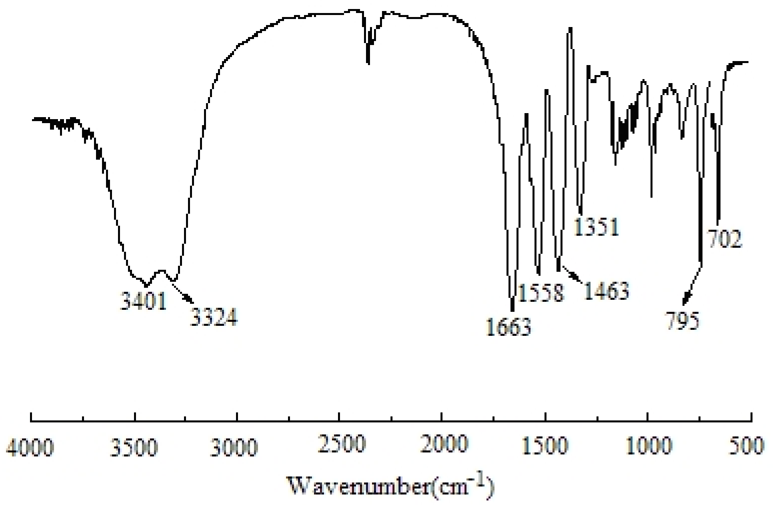

Fig. S6 FT-IR spectra of APBA

Analysis of PNIPAM-b-PAPBA

Table S1 showed the polymerization data of PNIPAM-b-PAPBA.

polymer	$M_{n,theo}$[a](g/mol)	$M_{n,\ GPC}$[b]	M_w/M_n[b]
$PNIPAM_{25}$	3180	3280	1.06
$PNIPAM_{25}$-b-$PAPBA_{27}$	7400	7620	1.24
$PNIPAM_{66}$	7700	8780	1.05
$PNIPAM_{66}$-b-$PAPBA_{20}$	11100	11600	1.22
$PNIPAM_{94}$	10900	11740	1.14
$PNIPAM_{94}$-b-$PAPBA_{21}$	13900	14880	1.39
$PNIPAM_{128}$	14800	15660	1.07
$PNIPAM_{128}$-b-$PAPBA_{19}$	17400	18500	1.35
$PNIPAM_{136}$	15600	16670	1.11
$PNIPAM_{136}$-b-$PAPBA_{16}$	18300	19200	1.37

Fig. S6 showed the M_n and M_w/M_n[b] of the PNIPAM-b-PAPBA.

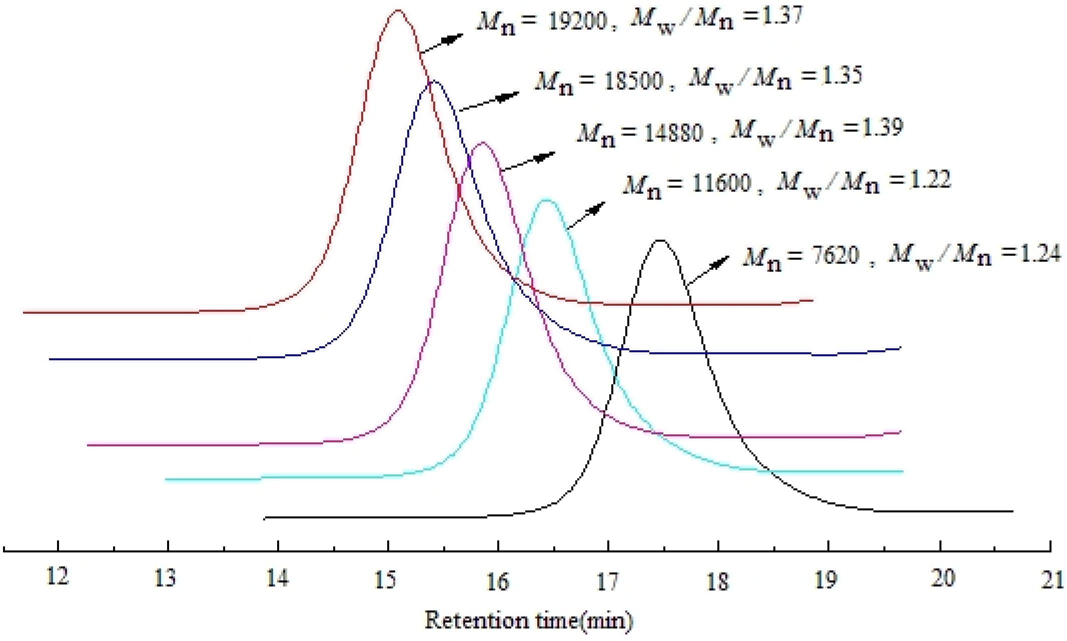

Fig. S6 GPC traces of PNIPAM-b-PAPBA use different macroCTA.

A ternary competitive complexation

Scheme S1 showed the competitive complexation of GA and glucose.

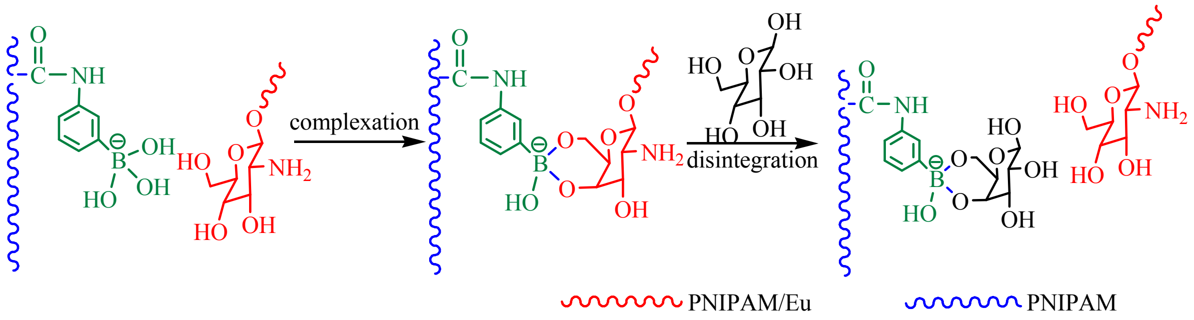

Scheme S1 Schematic illustration for the formation and glucose responsive disintegration of the complex micelles

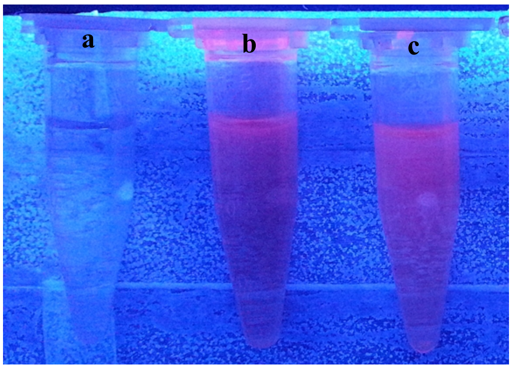

Fig.S7 The images for (a)the complexed micells of the PNIPAM-b-PAPBA and glucose,(b)dialysate,(c) the complexed micells at UV.

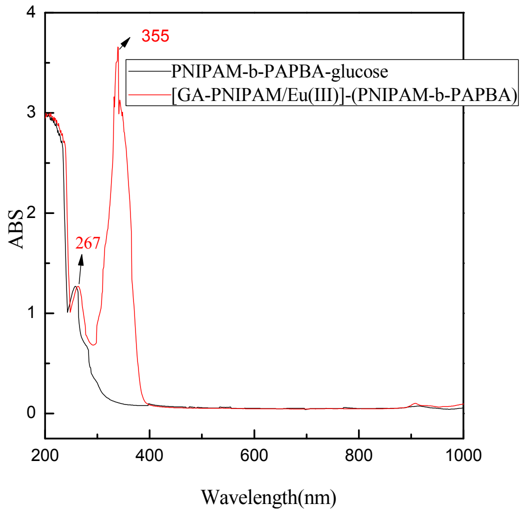

Fig. S8 UV-vis of the [GA-PNIPAM/Eu(III)]-(PNIPAM-b-PAPBA) and added the glucose.
